# Digging Further Into the Speech of Liars: Future Research Prospects in Verbal Lie Detection

**DOI:** 10.3389/fpsyt.2019.00056

**Published:** 2019-02-12

**Authors:** Galit Nahari, Zvi Nisin

**Affiliations:** ^1^Department of Criminology, Bar-Ilan University, Ramat-Gan, Israel; ^2^Investigative Psychology Section, The Division of Identification and Forensic Science, Investigation and Intelligence Branch, Israel Police, Haifa, Israel

**Keywords:** deception and deceptive strategies, verbal lie detection, verifiability approach, detection deception, reality monitoring, context embedded perception

The field of verbal lie detection has grown rapidly in the past decade. Derived by the assumption that lies have different content patterns than do truths, research in this area promotes searching for content criteria to detect them. One prime content-based indicator for deception detection, which stems from the Reality Monitoring (RM) theory (1), is *richness in detail*. According to RM, truthful memories of actual events originate in perceptual experience and are embedded in the context of time and space. As such, they are expected to include more spatial and temporal contextual attributes (i.e., locations, spatial arrangement of people and objects, times, duration and sequence of events) and perceptual attributes (i.e., what the individual felt, tasted, smelled, heard, or saw when the event took place) than do false memories, which originate in self-generated thought or imagination. Derived from this prediction, the traditional use of richness in detail as an indicator of deception is based on the *number* of perceptual and contextual details in the interviewee's accounts. However, as a memory source-monitoring theory, RM does not take into consideration the intention of liars to deceive and consequently cannot explain the full scope of *richness in detail* in the field of deception (2). In contrast to false memories, where the individual has no intention to deceive but *wrongly* believes that his/her memory of an event that never happened is truthful, fabricated memories are an outcome of manipulation [and have thus been labeled “self-manipulated memories”; (2)]. Liars frequently attempt to manipulate their fabricated accounts to make them seem truthful (3–5), for example by *intentionally* adding false perceptual and contextual details (6, 7). Affecting the quantity of the details in their fabricated accounts, such strategic manipulations reduce the diagnostic efficacy of the *richness in detail* indicator. Yet, in the current paper, we aim to show that the same strategies leave traces on the quality of details. Therefore, we propose that to maximize the potential utility of the *richness in detail* indicator, it is necessary to dig deeper into the speech of liars, particularly by looking for traces of deception strategies found in the *quality* of the details. In fact, the Verifiability Approach [VA; (4, 8)] applies this notion.

## The Verifiability Approach (VA)

The VA (8) for lie detection was initiated based on the understanding that lies, by nature, are based on strategies. The first VA study (4) clearly demonstrated that lie detection benefits more from consideration of the *quality* of perceptual and contextual details than it does from consideration of their quantity alone.

According to the VA, the strategy employed by liars is guided by the *liars' dilemma hypothesis*. Specifically, liars perceive richness in detail as an indicator of truthfulness (9, 10) and are thus motivated to provide many details to make an impression of honesty (7, 11). On the other hand, the provision of details also puts liars at risk, as the truthfulness of the details provided can be checked. Aware of this danger [see (6, 7)], liars are inclined to avoid mentioning false details, to minimize the chances of being caught. These two contradicting motivations—for and against the provision of details—put liars in a dilemma. A strategy that resolves the conflict involves the provision of details that cannot be checked and verified.

When used by liars, this strategy of providing non-verifiable information affects the quantity and quality of the contextual and perceptual details that appear in their accounts. They “inflate” the quantity of detail by incorporating false, non-verifiable, details, and as a result provide accounts that appear closer to the RM prototype of truthful accounts (i.e., accounts rich in perceptual and contextual details). However, their strategy leaves traces in the quality of their accounts, in terms of verifiability. By assessing the quality (i.e., the verifiability of the contextual and perceptual details) rather than the quantity of details provided, it is possible to reveal the liars' strategy, and thereby indicate their lies.

In the last years, the validity of the VA, which was originally developed and tested in police interview setting [e.g., (4, 5, 12, 13)] has been examined in other settings including insurance [e.g., (14–17)], airport security [see (18, 19)], occupation [e.g., (20)], and malingering [e.g., (21, 22)]. Some of these applications were more successful than others, but mostly the VA perspectives were confirmed [for a recent review see (23)], thereby providing an empirical evidence to the profitability of looking for quality of details. Encouraged by the success of the VA, we propose that research in this field should dig further into the speech of liars, in an attempt to identify additional indications of strategies in the quality of details provided. As such, we present two new approaches, both are derived from the theoretical and empirical framework of the VA.

## Context Embedded Perception (CEP)

The first approach was recently proposed by the authors of the current paper Nisin and Nahari (in preparation), who suggest that the qualitative differences between perceptual and contextual details can serve as a potential generator of deception strategy. According to this approach, while perceptual information is actually experienced, and acquired directly by the senses, contextual information is virtual in its nature, and based on semantic knowledge and relative conceptualizations. For instance, we experience the perceptual aspects of an interaction with a friend through our senses: we see the friend and the clothes he is wearing, hear his speech, and feel his touch. Meanwhile, the contextual aspects of this interaction, such as its length and specific location, are based on conceptualization and knowledge. In fact, the contextual attributes are imposed on the perceptual details and frame them in time and space. Accordingly, the perceptual details (e.g., visions, smells, sounds, sensations, and tastes) can be regarded as primary data, and the contextual details (e.g., indications of where, when, and for how long those perceptual details were experienced)—as meta-data. Obviously, the truthfulness of perceptual details can be checked only when they are given by the interviewee within the framework of contextual information regarding time and space. Thus, the contextual details are those that confer the status of verifiability upon perceptual details. Considering the differences between the two types of details in light of the VA (8), liars would be expected to avoid the provision of contextual details as often as possible. Motivated to provide non-verifiable details, they would be likely to provide perceptual details without framing them in time and space, making it difficult to check their truthfulness. Truth-tellers, on the other hand, would be expected to freely provide both types of details, as they have no reason to avoid verification. Thus, the prediction yielded from this approach is that liars, when adding false details to their accounts, will strategically prefer to provide perceptual details over (or without) contextual details, while truth-tellers will provide both types of details. As such, the number of contextual details in an account can serve as a verbal lie indicator.

## Resolution of Verifiability (RoV)

The second approach involves the resolution of the verifiable details provided, as determined by the immediacy in which the information they incorporate can be verify. A good example of such resolution involves the use of names, which already found significance for lie detection (24). According to the VA (4), events that occurred in the presence of another person will be considered verifiable only when that person can be traced. Once an identifiable person has been mentioned, that person can be approached to confirm the truthfulness of the reported occurrences. However, the mention of a name is not a necessary condition for rendering the person identifiable and traceable. It is reasonable to assume that an interviewee who mentions a “friend” or “neighbor,” even without volunteering a name, has considered that the police will ask about the person's specific identity, especially because identified persons can serve as witnesses (i.e., prime and significant evidence) who can confirm details in the interviewee's account. Consequently, it is likely that by mentioning persons who can be traced, with or without mentioning their names, interviewees mean, or at least are aware of the fact, that they are providing verifiable details [see (8)]. The difference between the two conditions (named vs. unnamed but traceable) is in the *resolution* of the verifiable details provided: names increase the resolution of the details. The mention of an identifiable person without a name leaves the interviewees with degrees of freedom, at least temporarily, such that the verifiability of the details is neither immediate nor easy (relatively speaking) to check. Importantly, these degrees of freedom also range, as mentioning an unnamed uncle, for example, leaves less degrees of freedom than mentioning an unnamed acquaintance. These assumptions lead to the expectation that liars, when they do provide verifiable details, will strategically prefer low-resolution over high-resolution details, while truth-tellers will prefer high-resolution details. Again, as with the contextual details, the number of low-resolution verifiable details in an account can serve as a verbal lie indicator.

## Conclusions

The current paper is a call for more strategy-based research in the field of verbal lie detection. By demonstrating how strategies blur the differences in *detail quantity* while sharpening the differences in *detail quality* between truthful and fabricated accounts, we stress that research should go beyond the surface of content, to look for strategies that activate verbal behaviors among liars, define the qualitative manifestations of their strategies, and then—to exploit these manifestations in indicating their lies. The VA, a new and promising paradigm for deception detection is an outcome of this research approach. Having presented two new, un-studied, strategy-based approaches to further demonstrate and extend this line of research, we propose that following this path will benefit the field both theoretically and practically.

## Author Contributions

GN: conceptualization and writing the first draft, revising and editing. ZN: conceptualization and review and editing.

### Conflict of Interest Statement

The authors declare that the research was conducted in the absence of any commercial or financial relationships that could be construed as a potential conflict of interest. The reviewer BK declared a past collaboration with one of the authors GN to the handling editor.

## References

[1] Johnson MK Raye CL Reality monitoring. Psychol Rev. (1981) 88:67–85.

[2] Nahari G The applicability of the verifiability approach to the Real world. In: Rosenfeld JP, editors. Detecting Concealed Information and Deception: Recent Developments. London: Elsevier (2018). p. 329–49.

[3] Granhag PA Hartwig M A new theoretical perspective on deception detection: on the psychology of instrumental mind-reading. Psychol Crime Law (2008) 14:189–200. 10.1080/10683160701645181

[4] Nahari G Vrij A Fisher R Exploiting liars' verbal strategies by examining unverifiable details. Legal Criminol Psychol. (2014) 19:227–39. 10.1111/j.2044-8333.2012.02069.x

[5] Nahari G Vrij A Fisher R The verifiability approach: countermeasures facilitate its ability to discriminate between truths and lies. Appl Cogn Psychol. (2014) 28:122–8. 10.1002/acp.2974

[6] Masip J Herrero C ‘What would you say if you were guilty?’ Suspects' strategies during a hypothetical behavior analysis interview concerning a serious crime. Appl Cogn Psychol. (2013) 27:60–70. 10.1002/acp.2872

[7] Nahari G Vrij A Fisher RP. Does the truth come out in the writing? Scan as a lie detection tool. Law Human Behav. (2012) 36:68–76. 10.1037/h0093965 22471387

[8] Nahari G Reality monitoring in the forensic context: digging deeper into the speech of liars. J Appl Res Mem Cogn. (2018) 7:432–40. 10.1016/j.jarmac.2018.04.003

[9] Bell BE Loftus EF. Trivial persuasion in the courtroom: the power of (a few) minor details. J Pers Soc Psychol. (1989) 56:669–79. 2724064 10.1037//0022-3514.56.5.669

[10] Johnson MK. Memory and reality. Am Psychol. (2006) 61:760–71. 10.1037/0003-066X.61.8.760 17115808

[11] Hartwig M Granhag PA Strömwall LA Guilty and innocent suspects' strategies during police interrogations. Psychol Crime Law (2007) 13:213–27. 10.1080/10683160600750264

[12] Nahari G Vrij A Are you as good as me at telling a story? Individual differences in interpersonal reality-monitoring. Psychol Crime Law (2014) 20:573–83. 10.1080/1068316X.2013.793771

[13] Nahari G Vrij A Can someone fabricate verifiable details when planning in advance? It all depends on the crime scenario. Psychol Crime Law (2015) 21:987–99. 10.1080/1068316X.2015.1077248

[14] Harvey CH Vrij A Leal S Lafferty M Nahari G. Insurance-based lie detection: enhancing the verifiability approach with a model statement component. Acta Psychol. (2017) 174:1–8. 10.1016/j.actpsy.2017.01.001 28088655

[15] Harvey AC Vrij A Nahari G Ludwig K Applying the verifiability approach to insurance claims settings: exploring the effect of the information protocol *Legal Criminol Psychol*. (2016) 22:47–59 10.1111/lcrp.12092

[16] Vrij A Nahari G Isitt R Leal S Using the verifiability lie detection approach in an insurance claim setting. J Investig Psychol Offend Profiling (2016) 13:183–97. 10.1002/jip.1458

[17] Nahari G Leal S Vrij A Warmelink L Vernham Z Did somebody see it? Applying the verifiability approach to insurance claims interviews. J Investig Psychol Offend Profiling (2014) 11:237–43. 10.1002/jip.1417

[18] Jupe LM Leal S Vrij A Nahari G Applying the verifiability approach in an international airport setting. Psychol Crime Law (2017) 23:812–25. 10.1080/1068316X.2017.1327584

[19] Kleinberg B Nahari G Arntz A Verschuere B An investigation on the detectability of deceptive intent about flying through verbal deception detection. Collabra: Psychol. (2017) 3:21 10.1525/collabra.80

[20] Jupe LM Vrij A Leal S Mann S Nahari G The lies we live: using the verifiability approach to detect lying about occupation. J Articles Support Null Hypothesis (2016) 13:1–13. Available online at: https://researchportal.port.ac.uk/portal/en/publications/the-lies-we-live(56beb3f2-0c7c-4821-905f-49bbba9c0b7e).html

[21] Boskovic I Bogaard G Merckelbach H Vrij A Hope L The verifiability approach to detection of malingered physical symptoms. Psychol Crime Law (2017) 23:717–29. 10.1080/1068316X.2017.1302585

[22] Boskovic I Gallardo CT Vrij A Hope L Merckelbach H Verifiability on the run: an experimental study on the verifiability approach to malingered symptoms. Psychiatry Psychol Law (2018). 10.1080/13218719.2018.1483272 [Epub ahead of print]. PMC6762097 31984064

[23] Vrij A Nahari G. The verifiability approach. In: Dickinson J Schreiber Compo N Carol R McCauley M, editors. Evidence-Based Investigative Interviewing. London: Routledge; Taylor and Francis Group (in press).

[24] Kleinberg B Mozes M Arntz A Verschuere B. Using named entities for computer-automated verbal deception detection. J Forensic Sci. (2018) 63:714–23. 10.1111/1556-4029.13645 28940300

